# The complete mitochondrial genome of a tea bagworm, *Mahasena colona* (Lepidoptera: Psychidae)

**DOI:** 10.1080/23802359.2017.1347839

**Published:** 2017-07-07

**Authors:** Pin-Wu Li, Shi-Chun Chen, Yi-Ming Xu, Xiao-Qing Wang, Xiang Hu, Ping Peng

**Affiliations:** a College of Horticulture, Sichuan Agricultural University, Sichuan, P.R. China;; b Tea Research Institute of Chongqing Academy of Agricultural Science, Chongqing, P.R. China

**Keywords:** Mitochondrial genome, *Mahasena colona*, tea bagworm

## Abstract

The mitochondrial genome of *Mahasena colona* Sonan has been sequenced and annotated completely. The entire genome is 16,119 bp in length with an A + T content of 82.85% (GenBank accession No. KY856825). The tea bagworm mt genome encodes all 37 genes that are typically found in animal mt genomes, consists of 13 protein-coding genes, 2 ribosomal RNA genes, and 22 transfer RNA genes. Within the mt genome of *M. colona*, there are six gene reading frame overlaps. The gene order is consistent with other sequenced mt genome of moths and butterflies in Ditrysia. The mt genome of *M. colona* contains a 728 bp A + T-rich region with a high A + T content of 97.66%.

The tea bagworm, *Mahasena colona* Sonan, belonging to the family Psychidae in the superfamily Tineoidea, occurs in China and India. It typically consumes tea leaves, influencing quality and quantity of tea products and causes considerable economic losses. In this study, larvae of *M. colona* were collected from a tea plantation in Rongchang, Chongqing, China, in June 2016, and identified to species by morphology. Voucher specimens (#CQNKY-LE-02-01-01) were deposited at the Insect Collection, Tea Research Institute of Chongqing Academy of Agricultural Science, Chongqing, China.

The complete mitochondrial genome of *M. colona* is shown to be a typical closed-circular and double-stranded DNA molecule in size of 16,119 bp (GenBank accession KY856825). It is high level among all reported Lepidoptera species and just smaller than *Formica fusca* (16,673 bp) (Babbucci et al. 2014), *Prays oleae* (16,499 bp) (van Asch et al. 2016), and *Thitarodes renzhiensis* (16,173 bp) (Cao et al. 2012). The overall nucleotide composition of the major strand of the tea bagworm mt genome as follows: A = 42.46% (6844), C = 10.46% (1686), G = 6.69% (1078), and T = 40.39% (6511), with a total A + T content of 82.85%, that is heavily biased toward A and T nucleotides. AT- and GC-skew of the whole J-strand of *M. colona* is 0.025 and −0.220, respectively.

The mt genome encodes all 37 genes usually found in animal mt genomes, including 13 protein-coding genes (PCG), 2 ribosomal RNAs, and 22 transfer RNAs. The gene arrangement in the mitochondrial genome of *M. colona* is conserved as other butterflies and moths mt genome in Ditrysia. In the mt genome of *M. colona*, a total of 26 bp overlaps have been found at six gene junctions of the genome (*atp8* and *atp6* share 7 nucleotides; *atp6* and *cox3* share a nucleotide; *trnN* and *trnS_1_* share 3 nucleotides; *nad4* and *nad4L* share 4 nucleotides; *trnI* and *trnQ* share 3 nucleotides; and *trnW* and *trnC* share 8 nucleotides). The mt genome is loose and has a total of 477 bp intergenic sequence without the putative A + T-rich region. The intergenic sequences are at 18 locations ranging from 1 to 98 bp, the longest one locates between *trnF* and *nad5*. The A + T-rich region of *M. colona* mt genome is 728 bp long and located between the *rrnS* and *trnM* genes. The A + T content of this region is 97.66%, the highest level of each region in this mt genome. This region includes the motif ‘ATAGA’ and a tandem repeat sequence with five 124 bp repeat units.

All 22 tRNA genes usually found in the mt genomes of insects are present in *M. colona*, 14 tRNA genes are encoded by J-strand and the others encoded by the N-strand. The nucleotide length of tRNA genes is ranging from 60 bp (*trnS_1_*) to 78 bp (*trnL_1_*), and A + T content is ranging from 73.24% (*trnK*) to 92.86% (*trnD*). 21 tRNA genes have the conventional cloverleaf shaped secondary structure and *trnS_1_* gene lacks the dihydrouridine (DHU) arm. The two rRNA genes have been identified on the N-strand in the *M. colona* mt genome: the *rrnL* gene locates between *trnL_1_* and *trnV* genes, and the *rrnS* gene between the *trnV* gene and the A + T-rich region. The length of *rrnL* and *rrnS* genes was 1440 bp and 792 bp, and their A + T content was 87.22% and 87.37%, respectively. The total length of all 13 protein-coding genes is 11,207 bp, which accounts for 69.53% of the whole genome sequence. The A + T content of the 13 genes ranges from 72.60% (*cox1*) to 87.01% (*atp8*). Twelve of the 13 PCGs start with ATN codons (ATG for *atp6*, *cox2*, *cox3*, *cob* and *nad4L*; ATT for *nad1-3* and *nad5*; ATA for *nad6* and ATC for *atp8*) and *cox1* used CGA as start codon, same situation exists in most Lepidoptera species (Chen et al. 2016). Two PCGs (*cox2* and *nad4*) have incomplete terminal codons consisting of single T nucleotide, and the other PCGs stop with TAA and TAG. The incomplete stop codon T is commonly reported and usually be confirmed by the tRNA punctuation model, also could produce functional stop codons in polycistronic transcription cleavage and polyadenylation mechanisms (Ojala et al. 1981; Boore 2001; Stewart and Beckenbach 2009).

We analyzed nucleotides sequences of 13 PCGs and 2 rRNAs with maximum likelihood (ML) method to understand the phylogenetic relationship of *M. colona* with other moths. The mt genome sequence of *Drosophila melanogaster* (GenBank accession no. DMU37541) was used as an outgroup. Both *M. colona* and *Tineola bisselliella* (Tineidae) belong to the superfamily Tineoidea and they are clustered into a branch of the phylogenetic tree with 92% bootstrap value (Figure 1). The suborder Ditrysia are monophyletic and Tineoidea are supported as the sister of the remaining Ditrysia (Timmermans et al. 2014).

**Figure 1.**
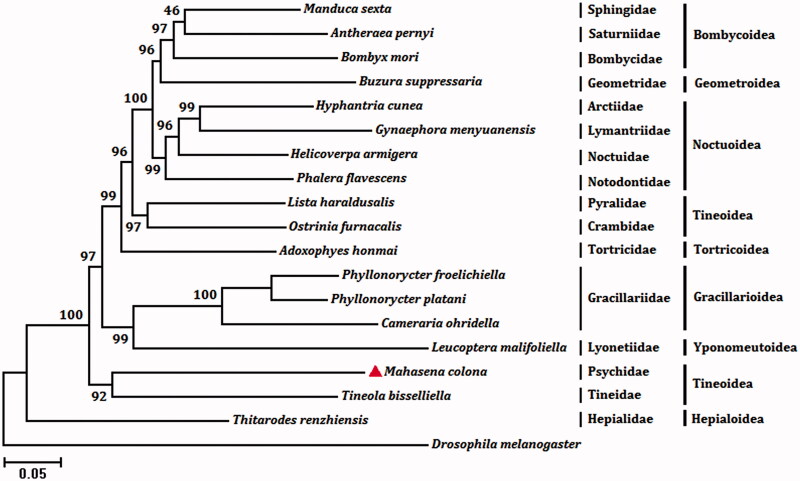
The maximum likelihood (ML) phylogenetic tree of *Mahasena colona* and other moths. The GenBank accession numbers used for tree constructed are as follows: *Tineola bisselliella* (KJ508045), *Cameraria ohridella* (KJ508042), *Phyllonorycter platani* (KJ508044), *Phyllonorycter froelichiella* (KJ508048), *Buzura suppressaria* (KP278206), *Bombyx mori* (AF149768), *Antheraea pernyi* (HQ264055), *Manduca sexta* (EU286785), *Thitarodes renzhiensis* (HM744694), *Helicoverpa armigera* (GU188273), *Phalera flavescens* (JF440342), *Hyphantria cunea* (GU592049), *Gynaephora menyuanensis* (KC185412), *Leucoptera malifoliella* (JN790955), *Adoxophyes honmai* (DQ073916), *Ostrinia furnacalis* (AF467260) and *Lista haraldusalis* (KF709449).
